# Intraflagellar Transport Proteins as Regulators of Primary Cilia Length

**DOI:** 10.3389/fcell.2021.661350

**Published:** 2021-05-19

**Authors:** Wei Wang, Brittany M. Jack, Henry H. Wang, Matthew A. Kavanaugh, Robin L. Maser, Pamela V. Tran

**Affiliations:** ^1^Department of Anatomy and Cell Biology, The Jared Grantham Kidney Institute, University of Kansas Medical Center, Kansas City, KS, United States; ^2^Department of Clinical Laboratory Sciences, The Jared Grantham Kidney Institute, University of Kansas Medical Center, Kansas City, KS, United States

**Keywords:** IFT-B, IFT-A, ciliogenesis, cilia disassembly, ectocytosis, posttranslational modification, kidney

## Abstract

Primary cilia are small, antenna-like organelles that detect and transduce chemical and mechanical cues in the extracellular environment, regulating cell behavior and, in turn, tissue development and homeostasis. Primary cilia are assembled via intraflagellar transport (IFT), which traffics protein cargo bidirectionally along a microtubular axoneme. Ranging from 1 to 10 μm long, these organelles typically reach a characteristic length dependent on cell type, likely for optimum fulfillment of their specific roles. The importance of an optimal cilia length is underscored by the findings that perturbation of cilia length can be observed in a number of cilia-related diseases. Thus, elucidating mechanisms of cilia length regulation is important for understanding the pathobiology of ciliary diseases. Since cilia assembly/disassembly regulate cilia length, we review the roles of IFT in processes that affect cilia assembly/disassembly, including ciliary transport of structural and membrane proteins, ectocytosis, and tubulin posttranslational modification. Additionally, since the environment of a cell influences cilia length, we also review the various stimuli encountered by renal epithelia in healthy and diseased states that alter cilia length and IFT.

## Introduction

Cilia or flagella are evolutionarily conserved organelles that protrude from a wide range of eukaryotic cells, from single-celled protists, like *Chlamydomonas reinhardtii*, to almost every vertebrate cell. Cilia are classified by function and structure into two general categories: motile and non-motile. Motile cilia not only generate motion but also contain receptors that provide sensory function (Jain et al., 2012). Motile cilia propel *C. reinhardtii*, as well as sperm, and sweep fluid and particles along the mammalian brain ventricles and respiratory and reproductive tracts. Non-motile cilia, also termed primary cilia, have chemo- and mechanosensory roles and are present on the sensory neurons of *Caenorhabditis elegans* and on most vertebrate cells. Primary cilia transduce light and mechanical and chemical cues (Poole et al., 1985; R Ferreira et al., 2019), mediate signaling pathways (Huangfu et al., 2003; Corbit et al., 2008; Wheway et al., 2018), and regulate cell cycle (Pan et al., 2013), cell differentiation (Ezratty et al., 2011; Forcioli-Conti et al., 2015), cell–cell communication (Viau et al., 2018), and autophagy (Pampliega et al., 2013; Orhon et al., 2016). The multiplicity and importance of these functions render primary cilia vital for organismal development and homeostasis (Badano et al., 2006).

**FIGURE 1:**
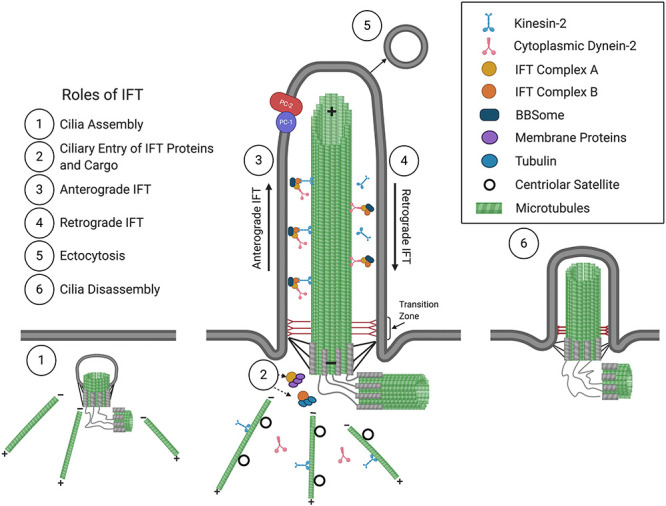
The primary cilium and roles of IFT in cilium assembly/disassembly. Consisting of a 9 + 0 arrangement of a microtubular axoneme ensheathed by a specialized ciliary membrane, the primary cilium assembles initially within the cytoplasm at the modified centriole (1), which becomes the basal body that forms the base of the primary cilium at the plasma membrane. Extension and maintenance of the cilium, along with the entry and exit of structural and functional components and the BBSome, are mediated by IFT (2–4). Roles for IFT in ectocytosis (5) and cilia disassembly (6) have also been implicated (see text for details). The positive (+) and negative (–) ends of axonemal and cytoplasmic microtubules are indicated. The polycystins, PC1 and PC2, are localized at the ciliary membrane and are mutated in ADPKD.

A cilium is comprised of a microtubule-based axoneme that extends from a modified centriole, the basal body, and is ensheathed by a ciliary membrane (Figure 1). In most motile cilia, nine doublet peripheral microtubules are arranged in a circle around two single central microtubules, while in motile cilia of the embryonic node as well as in non-motile cilia, only nine doublet peripheral microtubules comprise the axoneme, forming “9 + 2” and “9 + 0” conformations, respectively (Satir, 2005). Distal to the basal body is the ciliary gate, consisting of transition fibers that join the basal body to the base of the ciliary membrane, and the transition zone, comprised of modules and has Y-links that tether the most proximal part of the axoneme to the ciliary membrane. The transition fibers and transition zone regulate ciliary entry and exit of proteins, allowing for compartmentalization and formation of a unique environment, such that the cilioplasm and ciliary membrane are composed of proteins, second messengers, and phospholipids, distinct from that of the cytosol and plasma membrane (Deane et al., 2001; Stephan et al., 2007; Garcia-Gonzalo et al., 2011; Williams et al., 2011; Garcia-Gonzalo and Reiter, 2017; Gonçalves and Pelletier, 2017).

Primary cilia are dynamic structures that assemble and disassemble in coordination with the cell cycle. Cilia form when cells become quiescent, in G1 and G0, and begin disassembly before cells re-enter the cell cycle (Nigg and Stearns, 2011; Paridaen et al., 2013; Malicki and Johnson, 2017). The assembly and maintenance of cilia require intraflagellar transport (IFT), which was first observed in *C. reinhardtii* (Kozminski et al., 1993) and mediates the bidirectional transport of structural and signaling molecules along the microtubular axoneme. IFT is mediated by multiprotein complexes that can be dissociated biochemically into IFT complexes B (IFT-B) and A (IFT-A), consisting of 10 and 6 subunits, respectively. These IFT complexes form linear arrays or “trains” that are transported from the base to the tip of the cilium in anterograde IFT, powered by the kinesin-2 motor (Cole et al., 1998), then returned to the ciliary base in retrograde IFT, driven by cytoplasmic dynein-2 (Pazour et al., 1998, 1999; Porter et al., 1999; Signor et al., 1999). Another multiprotein complex, the BBSome, acts like an adaptor connecting IFT complexes to signaling molecules and is required for the ciliary export of activated signaling receptors (Nachury, 2018; Ye et al., 2018).

Primary cilia typically obtain a characteristic length for a cell type (Table 1), likely to achieve optimal function. In humans, mutation of ciliary genes results in disease syndromes, termed ciliopathies, which can manifest craniofacial defects, skeletal dysplasia, brain and cognitive defects, retinal degeneration, obesity, and fibrocystic disease of the liver, pancreas, and kidney (Badano et al., 2006; Hildebrandt et al., 2011). These mutations can cause signaling defects as well as cilia length differences (Bredrup et al., 2011; Halbritter et al., 2013; Alazami et al., 2014; Zhang et al., 2016; Duran et al., 2017; Shaheen et al., 2020). Additionally, in complex diseases and conditions not caused by a primary cilia genetic lesion, such as in obesity and type 2 diabetes and kidney injury, cilia lengths have also been reported to be shortened or lengthened on affected cells (Verghese et al., 2008; Han et al., 2014; Ritter et al., 2018; Yu et al., 2019). Thus, understanding cilia length regulation is critical to understanding the pathobiology of cilia-related disease.

**TABLE 1:** Mammalian cilia lengths.

**Cell type**			**Average cilia length, mm (range)**	**References**
Renal epithelia	Collecting ducts, 6 wks		∼4	Tran et al., 2014
	Tubules distal to the proximal tubule, P7		3.5 ± 1.7	Pazour et al., 2000
Cholangiocyte	5–12 months		3.26 ± 1.29	Stroope et al., 2010
Neuron	Hypothalamic arcuate nucleus, 21–30 weeks		∼3.5	Lee et al., 2020
	Hypothalamus	E12.5, E15.5	∼0.5	
		E18.5 P1	∼1	
		P7	∼1.5	
		P14	∼2	
		P28, P60	∼3.5	
	Hippocampal dentate gyrus, P14		∼2.8	
	Hippocampus, P15		∼3.2	
	Cerebellum, 3–5 months		∼3.8 (1–8)	Diaz et al., 2020
Neural tube	E9.5		∼1 (0.5–2)	Nandadasa et al., 2019
Osteocyte	*In vivo*	1 month	2.3 (1.5–3.4)	Lim et al., 2020
	*In vitro*	MLO-Y4 cells	∼2.8	Spasic and Jacobs, 2017
Osteoblast	*In vivo*	1 month	2.9 (1.4–4.3)	Lim et al., 2020
	*In vitro*	Primary osteoblasts, P0	∼2.6	Teves et al., 2015
		MC3T3-E1	3 ± 0.8 (1–5)	Li et al., 2020
Chondrocyte	*In vivo*	E16.5	1.20 ± 0.01	Martin et al., 2018
		P1	1.19 ± 0.02 (1–1.7)	Kunova Bosakova et al., 2018
		P3	∼1.6 (1–3)	
		P5	∼1.9 (1–3)	
	*In vitro*	Primary chondrocytes, E16.5	2.82 ± 0.05	Martin et al., 2018
		Primary chondrocytes, P0, P7	∼3	Teves et al., 2015; Liu et al., 2020
Endothelia	*In vitro*	Primary endothelial cells, E15.5	∼0.85	Abdul-Majeed and Nauli, 2011

*Values are of mouse tissues or cells. ∼ Indicates values were obtained from a graph within the publication. P, postnatal day; E, embryonic day; wks, weeks of age.*

Cilia length is determined by the balance of cilia assembly and disassembly (Marshall and Rosenbaum, 2001; Marshall et al., 2005). In *C. reinhardtii*, live imaging has revealed that frequency of IFT train ciliary entry, IFT train size and speed, and cargo loading vary with cilia length, which has led to various models of cilia length control (Marshall et al., 2005; Wren et al., 2013; Chien et al., 2017; Fai et al., 2019; Wemmer et al., 2020). However, primary cilia length regulation in mammalian cells has been much less studied. Here, we review the primary cilia phenotypes of IFT-B and IFT-A mammalian mutants to glean mechanisms by which IFT proteins influence cilia assembly/disassembly. This includes roles of IFT in ciliary trafficking of tubulin and membrane-associated proteins and in influencing ectocytosis. Since posttranslational modification of axonemal tubulin can promote cilia assembly or disassembly, we also review the effects of glutamylation and O-GlcNAcylation on IFT. In addition to these intrinsic ciliary factors, primary cilia lengths are modulated by changes in the extracellular environment. In the kidney, cilia lengths change in healthy and diseased states, including in polycystic kidney disease and during kidney injury and repair. Thus, we also review the effects of chemical and mechanical signals in the renal environment on cilia length and IFT.

## IFT in Ciliary Import of Structural and Membrane Proteins Affecting Ciliogenesis

To initiate ciliogenesis, the mother centriole matures into the basal body and migrates and docks at the plasma membrane. During migration of the mother centriole, preciliary vesicles derived from the Golgi and recycling endosome attach to the subdistal appendages of the maturing mother centriole and fuse into a larger ciliary vesicle (Sorokin, 1962). Centriolar coiled coil protein 110 (CP110) localizes to the distal end of the mother centriole and regulates the start of cilium extension (Chen et al., 2002; Yadav et al., 2016). Rab8a is recruited to the mother centriole and activated by Rab11 and Rabin8 to enable ciliary membrane assembly (Westlake et al., 2011). This, together with the recruitment of IFT and transition zone proteins to the cilia base, allows for cilium extension (Deane et al., 2001; Rosenbaum and Witman, 2002; Wang and Dynlacht, 2018).

### Ciliary Import of Tubulin

To extend the axoneme, α- and β-tubulin are imported into primary cilia and are added to the distal plus ends of microtubules at the cilia tip (Witman, 1975; Johnson and Rosenbaum, 1992). Live imaging of green fluorescent protein (GFP)-tagged tubulin in *C. reinhardtii* demonstrates that both diffusion and IFT allow for tubulin ciliary import (Craft et al., 2015; Craft Van De Weghe et al., 2020). In cells with growing cilia, anterograde transport of tubulin was increased, and in flagella length mutants, tubulin transport was dysregulated (elevated and reduced in *long flagella2-1* and *short flagella2* mutants, respectively, relative to steady-state wild-type cilia), suggesting a possible link between IFT-mediated transport of tubulin and regulation of cilia length (Craft et al., 2015; Wemmer et al., 2020).

In most IFT-B mutants, cilia are shortened or even lost (Table 2), indicating that the IFT-B complex is essential for ciliogenesis. The IFT-B complex consists of a 10-subunit core subcomplex and a 6-subunit peripheral subcomplex (IFT38, IFT57, IFT80, IFT20, IFT172, IFT54) (Katoh et al., 2016; Taschner et al., 2016). The core can be separated further into Core 1 (IFT25, IFT27, IFT74, IFT81, IFT22) and Core 2 (IFT56, IFT46, IFT52, IFT88, IFT70) subcomplexes (Nakayama and Katoh, 2020). Except for IFT74, all IFT-B components have been knocked out or mutated in mammalian cells with primary cilia (Table 2). Generally, loss of most Core 2 or peripheral subunits, with exception of IFT56, results in severely shortened or absent primary cilia. In contrast, deletion of Core 1 subunits has much milder effects, with many mutants lacking overt cilia length defects, although ciliary localization of signaling molecules and membrane-associated proteins is aberrant. Mammalian IFT70A and IFT70B constitute the orthologs of *C. reinhardtii* IFT70. Loss of both IFT70A and IFT70B in retinal pigment epithelial (RPE) cells causes the absence of cilia, while re-expression of either IFT70A or IFT70B restores ciliogenesis, revealing redundancy between IFT70A and IFT70B (Takei et al., 2018). In certain cases, cilia phenotypes are more severe in *C. reinhardtii* than in mammalian cells. For instance, IFT56-deficient *C. reinhardtii* have shortened cilia (Ishikawa et al., 2014), but *Ift56*-null mice do not (Xin et al., 2017). This may reflect greater functional redundancy among mammalian IFT proteins. Furthermore, deletion versus deficiency of an IFT protein can result in different ciliary phenotypes. Depletion of IFT80 in C3H10T1/2 mesenchymal cells causes a lack of cilia (Yang and Wang, 2012), while hypomorphic mutation of *Ift80* in mice results in normal cilia morphology (Rix et al., 2011), suggesting a threshold of IFT deficiency which can be tolerated.

**TABLE 2:** Mammalian IFT-B ciliary phenotypes.

**IFT-B**	**Cilia structure**	**Cell type**	**Cilia localization of proteins**	**References**
Core 1				
*Ift25* ko	No cilia length defects	MEF	Less GLI2 at ciliary tip, increased ciliary PTCH1 and SMO	Keady et al., 2012
*Ift27/Rabl4/Bbs19* ko	No cilia length defects	MEF, primary dermal fibroblasts	Increased ciliary SMO; diminished GLI2 at ciliary distal tip; decreased BBS and Arl6	Eguether et al., 2014; Liew et al., 2014; Yang et al., 2015
*Ift81* (deficiency)	Increased cilia length	Patient chondrocytes	None reported	Duran et al., 2016
*Ift81* loss-of-STOP mutation	Reduced ciliated cells and reduced number of cilia longer than 3 mm	Patient fibroblasts	No abnormalities in IFT or Hh protein ciliary localization but increased mRNA expression of *Gli2*	Perrault et al., 2015
*Ift22/Rabl5* ko	No cilia length defects	RPE		Takei et al., 2018
Core 2				
*Ift56*^*hop/hop*^ (null) or ko	No cilia length defects but reduced numbers of microtubule doublets and disrupted circular arrangement of microtubules	Neural tube, RPE	Reduced GLI2 and GLI3 at ciliary tip; no KIF17 at ciliary tip	Funabashi et al., 2017; Xin et al., 2017
*Ift46* ko	Loss of nodal cilia	Mouse node		Lee et al., 2015
*Ift52* (deficiency)	Shortened cilia but wider range of cilia lengths	Patient fibroblasts	Reduced IFT88	Zhang et al., 2016
*Ift88* ko	No cilia	Mesenchymal cells, renal epithelial cells, bone cells		Pazour et al., 2000; Haycraft et al., 2007
*Ift70A;Ift70B* dko	No cilia	RPE		Takei et al., 2018
Peripheral				
*Ift38/Cluap1* ko	No cilia	MEF, node		Botilde et al., 2013
*Ift57* deficiency	No cilia length defects	Patient fibroblasts	Altered ciliary distribution pattern of Ift57	Thevenon et al., 2016
*Ift80* ko	No cilia	C3H10T1/2 mesenchymal cells		Yang and Wang, 2012
*Ift80*^*gt/gt*^ hypomorph	No cilia length defects	MEF, renal epithelial cells	None reported but decreased *Gli1* and *Ptch1* mRNA expression in response to Hh agonist	Rix et al., 2011
*Ift20* strong kd/ko; mild kd	No cilia/less ciliated cells; no abnormalities	RPE, NIH/3T3	In mild *Ift20* kd, reduced ciliary polycystin 2	Follit et al., 2006
*Ift172*^*wim/wim*^; ko	No cilia or severely shortened	Mouse node, embryonic neuroepithelium		Huangfu et al., 2003; Gorivodsky et al., 2009
*Ift54/Trap3ip1^*GT/GT*^*	No cilia	MEF, neural tube		Berbari et al., 2011

*ko, knockout; dko, double ko; kd, knockdown; gt or GT, gene trap; MEF, mouse embryonic fibroblasts; RPE, retinal pigment epithelial cells; Hh, Hedgehog.*

Given that axonemal elongation requires anterograde transport of tubulin (Craft et al., 2015), the ciliogenesis defects of IFT-B mutants could result from the lack of IFT-based transport of tubulin. In *C. reinhardtii*, the N-termini of IFT74 and IFT81 dimerize and bind α- and β-tubulin as cargo of anterograde IFT (Bhogaraju et al., 2013; Kubo et al., 2016). Additionally, IFT proteins are designed to form protein–protein interactions, and the loss of an IFT-B subunit can cause destabilization of the IFT-B complex. The molecular architecture of the IFT-B complex appears conserved between *C. reinhardtii* and mammalian cells (Katoh et al., 2016). IFT88, together with IFT52, connects the IFT-B core and peripheral complexes (Taschner et al., 2016). In fibroblasts of a ciliopathy patient with short rib polydactyly, mutation of *IFT52* greatly reduced IFT52 protein levels, leading to a destabilized anterograde IFT complex, demonstrated by reduced cellular levels of IFT88, IFT74, IFT81, and ADP ribosylation factor like GTPase 13B (ARL13B), a ciliary membrane protein, as well as reduced IFT88 in cilia (Zhang et al., 2016). This anterograde IFT defect caused the presence of less ciliated cells and irregular distribution of ciliary lengths in patient cells.

Beyond the role of IFT-B subunits to form the IFT-B complex, IFT-B proteins also connect the IFT-B and IFT-A complexes, as well as anterograde and retrograde IFT. In *C. reinhardtii*, IFT74 was shown to associate IFT-B and IFT-A particles at the flagellar base and to be essential for flagellar import of IFT-A. Loss of the IFT74 residues required to bind IFT-A caused stunted cilia, thus revealing a role for the interdependence between IFT-B and IFT-A in ciliogenesis (Brown et al., 2015). Additionally, in mammalian retinal pigment epithelial (RPE) cells, expression of a truncated form of IFT88 on a CRISPR/Cas9-mediated IFT88 knockout background produced a ciliary phenotype similar to IFT-A knockout cells (Kobayashi et al., 2021). In *C. reinhardtii* and in mice, *Ift54*-null mutants lack cilia (Berbari et al., 2011; Zhu et al., 2017b), and a recent study showed that in *C. reinhardtii* and mammalian cells, IFT54 interacts with both the kinesin-2 and dynein motors (Zhu et al., 2020). Deletion of the IFT54 residues required to bind kinesin-2 reduced anterograde IFT, causing IFT motors and proteins to accumulate in the proximal region of cilia, while deletion of the residues that bind dynein impaired retrograde IFT, causing accumulation of IFT proteins at the distal tip (Zhu et al., 2020). Thus, IFT-B and IFT-A as well as anterograde and retrograde transport are interconnected, and these interconnections are integral to ciliogenesis.

### Ciliary Import of Membrane-Associated Proteins

The IFT-A complex consists of three core subunits (IFT122/IFT140/IFT144) and three peripheral subunits (IFT42/IFT121/IFT139) (Nakayama and Katoh, 2020). In mammals, the IFT139 homolog consists of two paralogs, THM1/TTC21B and THM2/TTC21A (Tran et al., 2008; Wang et al., 2020). With the exception of THM2, loss of any IFT-A core or peripheral subunit can result in shortened cilia with bulbous distal tips (Table 3). The severity of cilia phenotypes varies with cell type and/or *in vitro* or *in vivo* contexts. Additionally, as observed with IFT56, the occurrence of a more severe phenotype – loss of cilia – in *ift140*-null *C. reinhardtii* mutants (Picariello et al., 2019) compared to shortened cilia in mammalian cells (Liem et al., 2012; Hirano et al., 2017) supports that there may be greater redundancy among IFT proteins in mammalian cells or that other compensatory mechanisms exist.

**TABLE 3:** Mammalian IFT-A ciliary phenotypes.

**IFT-A**	**Cilia structure**	**Cell type**	**Cilia localization of proteins**	**References**
Core				
*Ift144*^*dmhd/dmhd*^ (null)	Extremely short cilia	Neural tube, MEF	No Arl13B, ACIII, or SMO entry; IFT-B accumulation	Liem et al., 2012
*Ift144* ko or kd	Slightly shortened cilia with bulbous distal tip	RPE	Loss of IFT-A around basal body, no entry of SMO or ARL13B; IFT-B, BBS4 accumulation	Fu et al., 2016; Hirano et al., 2017
*Ift144*^*twt/twt*^ hypomorph	No obvious length defects	Neural tube, MEF	Decreased ACIII; slightly increased GLI2 at ciliary distal tip	Liem et al., 2012
*Ift122^*sopb*/*sopb*^* (null)	Short with bulbous distal tip	Mouse node, MEF	Increased IFT-B, Gli2, Gli3 at distal tip; no TULP3	Qin et al., 2011
*Ift122* ko	No cilia	RPE	Loss of IFT-A and GPR161 around basal body, normal IFT-B around basal body; no SMO entry	Takahara et al., 2018
*Ift140 ^*cauli/cauli*^*	Short with bulbous distal tip	Limb buds		Miller et al., 2013
Peripheral				
*Ift121/Wdr35* mutant and ko	Shortened cilia with bulbous distal tip	Patient fibroblasts; RPE	Increased BBS4 and BBS5, Gli2, IFT-B, IFT-A, Kif3A; no ARL13B, INNP5E, SSTR3, MCHR1, serotonin receptor; reduced SMO	Fu et al., 2016; Duran et al., 2017
*Ift43* kd	Shortened cilia with bulbous distal tip	RPE	Increased IFT88, BBS4; no ARL13B	Fu et al., 2016
*Ift43* deficiency	Shortened cilia	Patient fibroblasts		Duran et al., 2017
*Thm1/Ttc21b^*aln/aln*^* (null) or kd	Shortened with bulbous distal tip	Limb bud, MEF, 3T3-LT1, renal epithelial	IFT-B, IFT-A, BBS, SMO accumulation at distal tip; decreased Arl13B, INNP5E, IFT-A	Tran et al., 2008; Tran et al., 2014; Jacobs et al., 2020; Wang et al., 2020
*IFT139/Ttc21b* ko	Normal cilia length with bulbous distal tip	RPE	IFT-B, IFT-A, SMO, GPR161 accumulation at distal tip	Hirano et al., 2017
*Thm2/Ttc21a*-null	Normal	MEF	Normal	Wang et al., 2020
Adaptor				
*Tulp3* kd; cko and K407I (deficiency)	No length defects	RPE; renal epithelia	Decreased SSTR3 and MCHR1; severely reduced ARL13B, reduced polycystin 2	Mukhopadhyay et al., 2010; Hwang et al., 2019; Legue and Liem, 2019
*Tulp3* ko	Shortened	RPE	No ARL13B or INNP5E	Han et al., 2019

*SSTR3, somatostatin receptor 3; Mchr1, melanin-concentrating receptor 1; ACIII, adenylate cyclase type III.*

Live imaging of short hairpin RNA (shRNA)-mediated *Thm1* knockdown inner medullary collecting duct (IMCD) cells expressing IFT88-eYFP demonstrates that a mammalian IFT-A subunit is required for retrograde IFT (Tran et al., 2008). Consistent with a general role for IFT-A in retrograde IFT, bulbous distal tips with sequestered IFT-B, IFT-A, BBS, and signaling proteins in mutants of both the IFT-A core and peripheral complexes indicate defective retrograde IFT (Table 3). Since retrograde IFT is required to bring back IFT proteins to the base for their recycling and re-entry into cilia, this could be a contributing mechanism by which IFT-A loss or deficiency decreases cilia assembly and, in turn, cilia length.

A comprehensive study examining ciliogenesis in *Ift121*/*Wdr35* knockout RPE cells showed that cilia assembly was reduced and delayed due to disruption of the ciliary import and export of the various cargoes of IFT-A (Fu et al., 2016). Rab8 localization near centrioles was reduced, suggesting that early formation of cilium membrane assembly is disrupted. Additionally, the localization of centriolar satellite proteins was misregulated. Centriolar satellites regulate protein composition of cilia and are essential for efficient ciliogenesis (Odabasi et al., 2019). Ciliary entry of ciliary membrane proteins, ARL13B and inositol polyphosphate-5-phosphatase E (INNP5E), was also impeded. This could contribute to impaired ciliogenesis, since ARL13B is essential for ciliary membrane extension, which is coupled to axoneme elongation (Lu et al., 2015). Ciliary ARL13B is also lost in *Ift144* knockout and *Ift43* knockdown cells and reduced in *Thm1*-null mouse embryonic fibroblasts (MEFs) (Liem et al., 2012; Fu et al., 2016; Wang et al., 2020). Furthermore, ARL13B was shown to bind to IFT43, IFT122, and IFT139/THM1, suggesting that ARL13B is a cargo passenger of IFT-A (Fu et al., 2016).

Fu et al. (2016) proposed a unifying mechanism for the IFT-A mutant defects in ciliary entry of membrane proteins and in retrograde IFT: that IFT-A mutation causes a defect in trafficking cargo to the minus ends of both axonemal and cytoplasmic microtubules. In *Ift122* knockout RPE cells, IFT-A localization around the basal body was lost, suggesting that IFT122 transports IFT-A proteins to the cilium base (Takahara et al., 2018). Similarly, in *C. reinhardtii*, loss of IFT43 resulted in reduced IFT proteins in the peri-basal body region, and both IFT43 and IFT140 were demonstrated to transport ciliary proteins from the cytosol to the peri-basal body region (Zhu et al., 2017a). These data support a defect in transporting cargo to the minus ends of microtubules with loss of IFT-A.

Depletion of IFT-A also causes ciliary localization defects of the transmembrane Hedgehog transducer, Smoothened (SMO). Loss of IFT-A core components, IFT144 and IFT122, results in absence of SMO in cilia (Liem et al., 2012; Hirano et al., 2017; Takahara et al., 2018), while loss of peripheral subunit IFT121 results in reduced SMO in cilia (Fu et al., 2016). In contrast, loss of THM1 causes increased ciliary SMO and its accumulation at the distal tip (Wang et al., 2020). While ciliary localization defects of SMO do not overtly affect cilia length, these differential phenotypes may reflect differences in cargoes or protein interactions of core versus peripheral subcomplexes or of individual IFT proteins. Like IFT121, THM1 is part of the peripheral subcomplex, and except for the opposing SMO ciliary localization defect, *Thm1*-null MEF show ciliary protein localization defects as well as reduced and delayed ciliogenesis (Wang et al., 2020), similar to *Ift121*-depleted RPE cells (Fu et al., 2016). However, while contrasting from other IFT-A mutants, the increased ciliary SMO in *Thm1*-null MEF could be due to a similar mechanism that causes increased ciliary SMO in *Ift25*- and *Ift27*-null IFT-B Core 1 mutants. IFT25 and IFT27 form a heterodimer and connect IFT to the BBSome, which exports signaling molecules out of primary cilia (Keady et al., 2012; Eguether et al., 2014; Liew et al., 2014).

In mammalian cells, the IFT-A core also binds to the tubby-related protein 3 (TULP3), which acts like an adaptor to import a subset of G protein-coupled receptors (GPCRs) into primary cilia. These GPCRs include melanin-concentrating hormone receptor (MCHR1), somatostatin receptor subtype 3 (SSTR3), GPR161, neuropeptide Y receptor 2 (NPY2R), and free fatty acid receptor 4 (FFAR4) (Mukhopadhyay et al., 2010, 2013; Badgandi et al., 2017; Hilgendorf et al., 2019). Consistent with the requirement of the IFT-A core for ciliary entry of TULP3, TULP3 was absent from cilia of an *Ift122*-null mutant mouse (Qin et al., 2011). *Tulp3* knockdown RPE cells, a *Tulp3* kidney-specific knockout mouse and a *Tulp3* hypomorphic mutant mouse showed a lack of ciliary ARL13B but normal cilia length (Mukhopadhyay et al., 2010; Hwang et al., 2019; Legue and Liem, 2019). However, *Tulp3* knockout RPE cells showed absent ciliary ARL13B as well as shortened cilia lengths (Han et al., 2019). Thus, cell specificity, extent of *Tulp3* depletion, and *in vitro* versus *in vivo* contexts, which would expose cilia to various extracellular factors, likely contribute to affecting cilia length. Unlike the loss of IFT-A core and peripheral subunits, *Tulp3* knockdown did not cause accumulation of ciliary IFT-B proteins (Mukhopadhyay et al., 2010), indicating Tulp3 is not required for retrograde IFT.

Since loss of many IFT-B proteins results in absence of primary cilia, the role of IFT-B in ciliary entry of membrane-associated proteins is comparatively less explored. IFT25 and IFT27, which are components of IFT-B Core 1, are dispensible for cilia formation but are required for ciliary removal of the BBsome and associated cargo (Keady et al., 2012; Eguether et al., 2014; Liew et al., 2014). Conversely, since ciliary entry of SSTR3 or SMO is not impeded in *Ift25*- or *Ift27*-null mutants, this may indicate that ciliary import of these receptors may not require IFT-B (Eguether et al., 2018; Ye et al., 2018). However, the role of other IFT-B proteins in SSTR3 and SMO ciliary import has not been investigated. Knockdown of *Ift57* and *Ift172* in IMCD cells severely reduced ciliogenesis, but in cells retaining primary cilia, D1-type dopaminergic receptors in the ciliary membrane was reduced, suggesting that IFT-B is required for ciliary import of certain membrane-associated signaling receptors (Leaf and Von Zastrow, 2015). This requirement for IFT-B in ciliary membrane import of D1-type dopaminergic receptors also involves Rab23 and Kif17. Supporting a role for IFT172 in associating with the membrane, *C. reinhardtii* IFT172 was shown to interact with and remodel membrane, and in mammalian (RPE) cells, IFT172 localized to the ciliary membrane (Wang et al., 2018).

## IFT in Modulating Ectocytosis

Primary cilia can release small vesicles or ectosomes, containing components from the ciliary membrane in a process termed ectocytosis (Long and Huang, 2019). This secretion function of cilia was first discovered in *C. reinhardtii* and now has been observed in multiple species, including in *C. elegans*, *Trypanosoma brucei*, and in mammalian cells (Wood et al., 2013; Wang et al., 2014; Long et al., 2016; Szempruch et al., 2016; Nager et al., 2017; Phua et al., 2017). Shedding of ciliary ectosomes or extracellular vesicles regulates ciliary compartmentalization and homeostasis, signaling, and organismal and intercellular communication.

There is some variation in the release of ciliary vesicles and their contents across organisms. In *C. reinhardtii*, ectosomes are released from the ciliary tip in a process that requires the endosomal sorting complex required for transport (ESCRT) pathway. *C. reinhardtii* ectosomes contain ciliary membrane proteins, enzymes, ubiquitinated proteins, and ESCRT proteins (Long et al., 2016). In *C. elegans*, sensory neurons release extracellular vesicles from the cilia base in a process that is ESCRT independent but requires IFT-B and IFT-A (Wang et al., 2014). These extracellular vesicles contain LOV-1 and PKD-2, the *C. elegans* polycystin orthologs, and regulate communication and mating-related behavior. In mammalian cells, ectocytosis occurs from the ciliary tip upon stimulation with growth factors, which changes the phospholipid content of the ciliary membrane via INNP5E (Phua et al., 2017). Mammalian ectosomes contain ciliary membrane proteins, GPCRs (Nager et al., 2017), as well as IFT-B proteins, which are not present in ciliary vesicles of *C. reinhardtii* and *C. elegans* (Phua et al., 2017). Ectocytosis is linked to cilia resorption and disassembly in *C. reinhardtii* and mammalian cells (Long et al., 2016; Phua et al., 2017), and the containment of IFT-B proteins in mammalian ectosomes may be a mechanism by which primary cilia dispose of IFT-B proteins and become primed for disassembly (Phua et al., 2017).

Studies with BBS and IFT mutants shed additional light on the regulation of ectocytosis. In IMCD cells null for regulators or subunits of the BBSome, such as IFT27, ARL6, or BBS2, failure of BBSome-mediated ciliary removal of activated GPCRs caused accumulation of active GPCRs at the ciliary tip, which was followed by ectocytosis (Nager et al., 2017). In *Thm1*-null MEF, which sequester proteins at the ciliary tip, serum stimulation following starvation caused an increased presence of IFT-B foci that were separate from and distal to the cilia tip (Wang et al., 2020). Such observations likely reflect ectocytosis. In RPE cells depleted of mitogen-activated protein kinase-like kinase, ICK, which binds to IFT-B and localizes at ciliary tips, cilia were lengthened with an accumulation of IFT and signaling proteins at the distal tip, indicative of impaired retrograde IFT. Furthermore, these accumulated proteins were released in extracellular vesicles at the distal tip, indicative of ectocytosis (Nakamura et al., 2020). Collectively, these studies suggest that defective retrograde IFT promotes ectocytosis.

Ectocytosis precedes cilium resorption and disassembly (Phua et al., 2017). Consistent with this pattern, *Thm1*-null MEF, which exhibit a phenomenon consistent with increased ectocytosis also show enhanced serum-induced cilia loss (Wang et al., 2020). Cilia disassembly can occur via cilia resorption, which shortens cilia length gradually; complete cilia shedding, which severs the entire cilium; and a combination of both. Complete cilia shedding was found to be the predominant mechanism of cilia disassembly in wild-type mammalian cells (Mirvis et al., 2019). Future investigation into the role of IFT in regulating cilia disassembly could deepen our understanding of why one mechanism prevails over another.

## Tubulin Posttranslational Modification and IFT

Posttranslational modification (PTM) of the axonemal microtubules affects their stability, promoting either cilia assembly or disassembly. Acetylation, glutamylation, and glycylation stabilize the axoneme, favoring cilia assembly (Pugacheva et al., 2007; Shida et al., 2010; Gadadhar et al., 2017; He et al., 2018). In contrast, ubiquitination destabilizes the axoneme, leading to cilia disassembly (Huang et al., 2009; Wang et al., 2019).

Studies also suggest that PTMs regulate IFT. In shortening flagella of *Chlamydomonas*, IFT139 was identified to be ubiquitinated and to interact with ubiquitylated α-tubulin, suggesting that the IFT-A complex may traffic ubiquitylated axonemal proteins out of flagella, thus contributing to cilia disassembly (Wang et al., 2019). In *C. elegans*, a *ccpp-1* deglutamylase mutant altered stability of the axonemal β-tubules and increased velocity of the kinesin 3 KSP-6 and kinesin 2 SOM-3/KIF17 accessory motors (O’hagan et al., 2011). Also in *C. elegans*, starvation activated tubulin glutamate ligase 4 (TTLL4), which increased glutamylation, as well as the speed of the kinesin II canonical motor and, hence, of anterograde IFT (Kimura et al., 2018).

Recently, N-acetylglucosamine (GlcNAc), a nutrient sensor and product of the hexosamine biosynthetic pathway that can be linked enzymatically to serine/threonine residues of a multitude of intracellular proteins (Hart et al., 2011), has been shown to regulate cilia length (Tian and Qin, 2019; Yu et al., 2019, 2020). Both tubulin and histone deacetylase 6 (HDAC6, which regulates cilia disassembly) (Pugacheva et al., 2007), are O-GlcNAcylated (Tian and Qin, 2019). In one study, pharmacological and genetic inhibition of O-GlcNAc transferase (OGT), which transfers the GlcNAc moiety onto protein substrates, increased cilia length in RPE and IMCD cells (Tian and Qin, 2019). In another study, tissues (retinal photoreceptors, trachea) of *Ogt* knockout mice and RPE cells treated with an OGT inhibitor displayed shortened primary cilia with an accumulation of IFT proteins in bulbous distal tips, suggesting impaired retrograde IFT (Yu et al., 2020). While more investigations are required to reconcile these opposing results, these studies indicate that O-GlcNAcylation is another PTM affecting cilia length and IFT.

Together, these studies hint at a role for IFT in mediating the cilium assembly/disassembly associated with a particular PTM. Live imaging of IFT in mammalian cells following a PTM event is required to establish that PTMs affect IFT and to explore how PTMs regulate the IFT machinery.

## Modulation of Renal Epithelial Cilia Length and Renal Function

In a context-dependent manner, primary cilia demonstrate multiple roles. These organelles sense and transduce light, chemical, and mechanical cues and are hubs for multiple signaling pathways, including Hedgehog, Wnt, Notch, transforming growth factor beta (TGFβ), platelet-derived growth factor receptor (PDGFR), and G protein coupled receptor (GPCR) (Huangfu et al., 2003; Schneider et al., 2005; Eggenschwiler and Anderson, 2007; Corbit et al., 2008; Ezratty et al., 2011; Clement et al., 2013; Anvarian et al., 2019). Activation or downregulation of these signaling pathways regulates cell behavioral response, including proliferation, differentiation, and tissue organization. Mutation of ciliary genes reveals importance of primary cilia in multiple organ systems. The presence or absence as well as severity of phenotypes or clinical manifestations reflect the cell-specific and developmental and homeostatic roles of primary cilia.

Among the first revelations that primary cilia are essential to mammalian health was the discovery that mutation of IFT causes renal cysts (Yoder et al., 1995, 2002; Pazour et al., 2000). In renal tubules, cilia extend from the apical surface of most epithelial cells, suggesting that the role of renal tubular epithelial cilia is to sense the environment of the renal tubular lumen and transmit this information into the epithelium and possibly to “downstream” tubule segments (via ectosomes). Analyses of human kidneys reveal that cilia lengths change with nephron segment, as well as with development, being shortest in renal vesicles (0.59 μm) and lengthening as fetal nephrons mature (3.04 μm) (Saraga-Babic et al., 2012). Additionally, mutation of genes required for ciliogenesis usually causes shortened or absent cilia, and less commonly lengthened cilia, and often results in fibrocystic renal disease (Davis et al., 2011; Srivastava et al., 2017). In mice, the mutation and/or deletion of IFT genes models this phenomenon (Yoder et al., 1995, 2002; Pazour et al., 2000; Jonassen et al., 2008, 2012; Patel et al., 2008; Tran et al., 2014). Together, these findings indicate that the presence and architecture of primary cilia regulate kidney development, morphology, and function.

In autosomal dominant polycystic kidney disease (ADPKD), the majority of mutations occur in *PKD1* and *PKD2*, which encode polycystin 1 and polycystin 2, which form a complex and function at the primary cilium (Freedman et al., 2013; Cai et al., 2014; Walker et al., 2019). In renal tissue of ADPKD patients as well as of orthologous mouse models, cilia lengths are increased (Hopp et al., 2012; Liu et al., 2018; Shao et al., 2020). Similarly, in the *jck* non-orthologous mouse model of ADPKD, renal primary cilia are also lengthened (Smith et al., 2006). Interestingly, deletion of *Kif3a*, *Ift20*, and *Ift88* in *Pkd1* and *Pkd2* conditional knockout mice ablated or shortened primary cilia and markedly attenuated the ADPKD cystic phenotype (Ma et al., 2013; Viau et al., 2018; Shao et al., 2020). Similarly, pharmacological shortening of primary cilia (via a CDK5 inhibitor) in *jck* mice ameliorated the cystic disease (Husson et al., 2016). Conversely, genetic and pharmacological inhibition of cilia disassembly in *Pkd1* conditional knockout mice increased renal cilia lengths and worsened ADPKD severity (Nikonova et al., 2014). These data suggest that cilia length may be an important modifier of ADPKD severity.

Renal epithelial cilia lengths also change during acute and chronic kidney injury and repair. A variety of methods have been employed to induce renal injury in both *in vitro* and *in vivo* model systems (Table 4). Acute injury, or the exposure to hypoxia to model chronic injury, appears to cause deciliation followed by elongation of cilia and eventual return to normal cilia length. Such alterations in cilia length are proposed to alter cilium sensing function and to form part of an epithelial repair mechanism whereby cilia regulate changes in signaling pathways appropriate for normal repair of injured renal tubules (Verghese et al., 2019). This dynamic modulation of renal cilia lengths during renal injury and repair suggests that cilia lengths are tuned to maintain normal renal tubular structure and function (Verghese et al., 2008, 2009; Han et al., 2017; Park, 2018).

**TABLE 4:** Modulators of renal cilia length.

	**Cilia length**	**Cell type**	**Mechanism or cellular effect**	**References**
*In vitro*				
↓ intracellular Ca^2+^	↑	IMCD, MEK, BME	Increased anterograde IFT speed	Besschetnova et al., 2010
↑ intracellular cAMP	↑	IMCD, MEK, BME	Activated PKA; increased anterograde IFT speed	Besschetnova et al., 2010
Fluid flow	↓	IMCD, MEK, BME	↓ cAMP; decreased cAMP and cilia length response abolished by *Pkd1* and *Pkd2* kd or mutation	Besschetnova et al., 2010
H_2_O_2_ (reactive oxygen species)	Deciliation	MDCK		Kim et al., 2013
Dopamine	↑	LLC-PK1	Increased cilia length increases intracellular Ca^2+^ response to fluid shear stress	Upadhyay et al., 2014
Cobalt chloride (hypoxia)	↑	MDCK	Increased HIF1-alpha	Verghese et al., 2011
aldosterone	↑	mCCD	Reduced degradation of IFT88	Komarynets et al., 2020
*In vivo*				
*Pkd1*^*RC/RC*^; *Pkd1* ko or *Pkd2* ko; *Pkd1^*RC/RC*^:Pkhd1* ko	↑	Collecting duct; cyst-lining renal epithelia		Hopp et al., 2012; Liu et al., 2018; Olson et al., 2019
*Nek8*^*jck/jck*^ mutant	↑	Cyst lining renal epithelia		Smith et al., 2006
Ischemia	↓ 1 day after; ↑ 8 days after	Bowman’s capsule parietal epithelium, proximal tubule, collecting duct	Deciliation	Verghese et al., 2008; Kim et al., 2013
*RBPj* ko (Notch signaling downregulation)	↑	Cystic renal epithelia of developing kidney	Increased Akap12, a scaffolding protein that interacts with PKA, cyclins, and protein kinase C	Mukherjee et al., 2020
Hypoxia (chronic renal injury)	↑	Fetal ovine renal epithelia	Increased cilia length increased intracellular Ca^2+^ response to fluid shear stress	Shamloo et al., 2017
Mineralocorticoid receptor ko	↓	Distal nephron	Inability to respond to aldosterone	Komarynets et al., 2020

*IMCD, mouse inner medullary collecting duct cells; MEK, mouse embryonic kidney cells; BME, bone mesenchymal cells; MDCK, Madin–Darby Canine Kidney cells; LLC-PK1, Lilly Laboratories Cell-Porcine Kidney 1 cells; mCCD, murine cortical collecting duct cells.*

Cellular signaling pathways and signals from the local environment modulate cilium length. As renal primary cilia protrude into the tubular lumen, cilia are exposed to filtrate flow. In response to fluid flow, primary cilia of cultured renal epithelial cells deflect, and intracellular calcium (Ca^2+^) increases (Praetorius and Spring, 2001, 2003). In cells that are stimulated resulting in lengthened cilia, the increase in intracellular Ca^2+^ induced by fluid flow is elevated, indicating that increased cilia length increases sensitivity to fluid flow (Upadhyay et al., 2014; R Ferreira et al., 2019). Conversely, in cells mutant for *Pkd1* or *Pkd2*, the increased intracellular Ca^2+^ response to fluid flow is abrogated (Nauli et al., 2003). In ADPKD cells, intracellular Ca^2+^ is decreased, and intracellular cyclic AMP (cAMP) is increased (Yamaguchi et al., 2004). Treatments to decrease intracellular Ca^2+^ and to elevate intracellular cAMP of cultured IMCD cells increased primary cilium lengths (Besschetnova et al., 2010). Using live microscopy, the increase in cilia length coincided with increased velocity of anterograde IFT. Additionally, fluid shear-mediated deflection reduced cilia length, decreased intracellular cAMP, and reduced the intracellular Ca^2+^ response to fluid flow, indicating the presence of a negative feedback loop. In *Pkd1*- or *Pkd2*-deficient cells, the Ca^2+^ response to fluid flow was abolished, and so was the negative feedback loop. In the absence of fluid flow, cultured *Pkd1* MEF and renal epithelia also displayed reduced cilia disassembly via disruption of a centrosomal integrity pathway mediated by p53 (Gerakopoulos et al., 2020). In contrast, Streets et al. reported shortened primary cilia in *Pkd1*-mutant renal epithelial cells via increased actin polymerization mediated by RhoA (Streets et al., 2020). These varied results may reflect that the altered cilia lengths are captured at a particular time point during dynamic modulation of cilia length by these individual pathways. Together, these studies demonstrate that regulation of cilia length and cilia-mediated function is tightly connected. Furthermore, the disruption of this connection in ADPKD suggests that this connection may be critical to staving off disease.

Extrinsic factors that regulate kidney homeostasis have also been shown to alter cilia length. Dopamine activates the intrarenal dopaminergic pathway to reduce salt and water reabsorption by the kidney, and dysregulation of intrarenal dopaminergic signaling increases risk for essential hypertension (Harris and Zhang, 2012). Treatment of LLC-PK1 porcine kidney epithelial cells with dopamine and fenoldopam to activate ciliary-localized dopamine receptor type-5 increased cilia length (Upadhyay et al., 2014). Fenoldopam caused a greater increase in cilia length than dopamine, and increased cilia length correlated with the sensitivity of the cells to fluid shear stress as measured by the increase in intracellular Ca^2+^, substantiating the interconnection between cilia length modulation and ciliary signaling. Aldosterone, a mineralocorticoid steroid hormone that is produced by the adrenal cortex, conserves sodium in the kidney. In a mineralocorticoid knockout mouse, cilia of multiple tubule types were shortened. Explaining this result, treatment of cultured cortical collecting duct cells with aldosterone increased primary cilia length, and this increase correlated with intensity of the transepithelial Na+ transport and was demonstrated to occur via reduced degradation of IFT88 (Komarynets et al., 2020). Thus, modulation of cilia length is inherent to kidney homeostasis and physiology.

## Discussion

Primary cilia lengths differ with cell type, developmental stage, disease state, and in response to injury or repair. Multiple intrinsic (IFT and ciliary proteins) and extrinsic factors contribute to modulating cilia length, and these factors are likely intertwined to control cilia length and function. More analyses in mammalian cells are required to determine the extent to which mechanisms discovered in *C. reinhardtii* and other ciliated organisms are similar or diverge in mammalian cells. The redundancy of IFT proteins in mammalian cells suggests a means of additional species- or cell-specific roles for these proteins in regulating cilia length and function. High-resolution live microscopy of IFT and associated cargo in mammalian cells will be instrumental in obtaining new mechanistic insights.

Signaling events can affect cilia length, which in turn influences sensitivity to external factors, such as fluid flow in the kidney. Expanding our knowledge of the signaling pathways that influence cilia lengths and those that are affected by cilia length would contribute to a regulatory network of cilia length control. Since studies suggest that cell type and *in vitro* versus *in vivo* contexts can produce differential results, expansion of cell types and development of *in vitro* models that more closely mimic the *in vivo* setting, such as 3D models and cultures that implement multiple cell types, may help to reconcile these differences.

Given that rescue of the increased cilia length in ADPKD mouse models correlates with attenuation of the disease, could cilia length in certain cases be a therapeutic target? In a developmental study, the rescue of cilia length and structure of *Dync2h1*-null embryos via deletion of one allele of *Ift172* mitigated the Hh signaling defects and early embryonic lethality, suggesting that *Ift* mutations cause their phenotypes primarily by affecting cilia architecture rather than by directly regulating signaling (Ocbina et al., 2011). Since *Ift* genes have differential roles, determining how to rescue cilia architecture for each *Ift* gene deletion would require expanding digenic analyses to multiple *Ift* genes. Additionally, cancer studies show that drugs can have differential effects on cilia lengths of different cell types within a tissue, emphasizing cell-specific control of ciliogenesis (Kiseleva et al., 2019).

However, in ciliopathy patient cells, cilia length is not always altered, or inconsistencies exist in whether a gene mutation causes cilia to be lengthened or shortened. This could suggest that clinical manifestations are due to signaling defects and that cilia length alterations *per se* may not be the primary cause of perturbed signaling. Rather, IFT proteins may regulate both cilia architecture and signaling pathways. Technologies to target a signaling pathway of a specific cell (Shillingford et al., 2012) or cilia (Pala et al., 2019a, b) of a particular cell type may further help provide the molecular tools to treat cilia-related disease.

## Author Contributions

WW, RM, and PT wrote the first draft. HW constructed Figure 1. WW, BJ, HW, MK, RM, and PT edited the manuscript and approved the final version.

## Conflict of Interest

The authors declare that the research was conducted in the absence of any commercial or financial relationships that could be construed as a potential conflict of interest.
